# Addressing the Fortification Quality Gap: A Proposed Way Forward

**DOI:** 10.3390/nu12123899

**Published:** 2020-12-20

**Authors:** Laura A. Rowe

**Affiliations:** Food Fortification Initiative, Atlanta, GA 30322, USA; laura.rowe@ffinetwork.org

**Keywords:** fortification, micronutrients, micronutrient deficiency, large-scale food fortification, monitoring, regulatory monitoring, compliance, fortification quality, premix reconciliation calculation

## Abstract

Large-scale food fortification is an effective, sustainable, and scalable intervention to address vitamin and mineral deficiencies, however, pressing gaps exist globally around ensuring the quality of fortified foods. This paper summarizes the global challenges and gaps faced in monitoring the quality of fortified foods, the guidance produced in response to these challenges, where we are today in terms of effective implementation, and what approaches and opportunities may be usefully applied to enhance the quality of fortified foods moving forward.

## 1. Introduction

Nutritionally adequate diets continue to be threatened in many parts of the world, including in low-, middle-, and high-income settings. This is due to a myriad of factors, including food systems that do not contain nutritious foods; limited purchasing power of individuals and families to purchase nutritious foods; limited access to nutritious foods and markets [1]; and that food systems don’t make nutritious foods convenient or desirable. These limitations can result in significant deficiencies in essential vitamins and minerals such as iron, folic acid, zinc, vitamin A, and iodine, among others, and have devastating and often irreversible consequences for individual health and national economies [2,3].

The fortification of food with vitamins and minerals is widely recognized as a cost-effective and sustainable strategy to improve the nutritional health status of populations [4,5,6] and is a unique health intervention, in that it leverages the expertise and delivery platform of the private food sector, relieving often overburdened healthcare systems. As a result, fortification has the potential to reach very large portions of the population and is an important complement to other nutrition-specific and nutrition-sensitive interventions. 

There is strong evidence that food fortification has led to significant positive impacts on nutritional status [7]. A recent systematic review and meta-analysis of large-scale food fortification programs found that, when implemented population-wide, these programs were associated with a 34% reduction in anemia from improved iron stores, a 74% reduction in the odds of goiter due to increased iodine intake, and a 41% reduction in the odds of neural tube defects (NTDs) as a result of increased folate intake [8]. 

Despite these successes however, food fortification programs, globally, are at risk of not reaching their full potential [9]. Although there are numerous reasons why this is the case, from countries that could benefit from fortification that do not have programs in place to existing programs that are designed, implemented, and operated sub-optimally [9,10,11,12], a significant roadblock to effective programming has proven to be collecting and acting upon national fortification monitoring information. Obtaining data (and ensuring that data is of good quality) on whether or not foods are actually fortified according to a country’s national fortification standard (that is whether or not they contain the designated amount of vitamins and minerals) and, subsequently, taking action based on that data to limit the number of non-fortified or under fortified products on the market, has proven to be a near universal challenge for countries implementing large-scale fortification programs [13,14,15].

In this paper, first, an outline is provided of countries that have national-level information on fortification quality and how that information was collected based on data available through the Global Fortification Data Exchange (GFDx). Second, documented challenges in collecting and acting upon fortification compliance or quality data are collated, based on published studies and recent content-specific workshops. Third, a brief summary is provided of recently developed tools that address fortification quality challenges. Fourth, an under-utilized but potentially effective means of more realistically acquiring fortification quality information is provided, with examples of countries that have (successfully and unsuccessfully) attempted to use this approach. Finally, with an understanding of these constraints, a way forward is suggested for program implementors and policy makers to effectively gain a better picture of national fortification quality, and thereby enhance the potential impact of fortification programs. 

## 2. Background on Regulatory Monitoring and Compliance of Food Fortification Programs 

Regulatory monitoring of fortified food, in the context of mandatory fortification programs, requires tracking indicators, such as the quality and quantity of fortified food produced and imported over time, to ensure programs stay on track to achieve the desired aim of increasing the intake of essential vitamins and minerals in a population [14]. Regulatory monitoring is the primary means by which compliance with food standards, including fortification standards, is determined [16,17]. Regulatory monitoring of fortified foods can take place at four different levels:

1. Internally at food production facilities—here food producers are expected to put in place internal quality assurance and quality control protocols that allow for regular checking and adjusting of the fortification production process, which should be built into already-existing food safety and quality measures, such as Hazard Analysis Critical Control Point (HACCP) and Good Manufacturing Practices (GMP) measures;
2. Externally by government food control agencies who are expected to verify that food producers have appropriate quality assurance and quality control measures in place that are well documented via technical audits and, occasionally, qualitative or quantitative end product tests. (Technical audits compliment the internal monitoring procedures that food manufacturers regularly implement and track. During an audit, an inspector will verify the production procedures and ensure documentation of the procedures are in place by observing the fortification process, conducting critical location checks (e.g., inside the feeder and inside the premix storage area), confirming that quality assurance and quality control protocols are established and followed, and reviewing records that document internal monitoring practices [18]. Verification of procedures and documentation is generally a more effective way of ensuring the end product is of adequate quality than end product tests.);
3. At the import level by government customs or food control agencies, who are expected to check to be sure fortified foods imported into the country meet national standards—this can include verifying certificates of analysis and, occasionally, conducting qualitative or quantitative tests; and
4. At the market level by government food control agencies, who are expected to verify fortified products’ packaging and labelling; this may occasionally include qualitative or quantitative testing [19] to gauge fortification program performance.

Throughout this paper, the terms “fortification quality” and “fortification compliance” will be used interchangeably.

Data collected through regulatory monitoring activities is vital to understanding how compliance with fortification requirements is being achieved, and if and where adjustments need to be made to ensure the program can deliver its intended nutritional benefits. This has proven, however, to be one of the most difficult and complicated pieces of a fortification program to implement and sustain. There is little value in measuring the health impact of fortification programs if food producers are not able to determine whether foods are fortified at levels stipulated in the national standard, or if government inspectors are not able to confirm this data from food producers. Therefore, having a complete and comprehensive understanding of on-the-ground constraints related to monitoring the quality of fortification programs is vital. How to ensure monitoring requirements and expectations are in line with what can realistically be carried out is outlined below. The design of such programs and the stakeholders that should be involved is not the subject of this paper. 

### 2.1. Current Status of National Fortification Program Rates of Compliance

The Global Fortification Data Exchange (GFDx) is an online resource that aggregates and visualizes data on five commonly fortified foods: maize flour, wheat flour, oil, rice, and salt. All data in the GFDx comes from reports from countries and national programs. 

GFDx [20] uses three categories of fortification quality information: compliance, quality, and expert opinion. “Compliance” data refers to industry compliance data obtained by authorized government entities on production volumes, market share, or samples and facilities monitored. “Quality” data is proxy data for estimating fortification quality obtained from market or household (HH) samples analyzed by non-governmental entities. “Expert opinion” data is proxy data for estimating fortification quality based on an individual’s belief or understanding of what may exist in the country. The more reliable means of obtaining fortification monitoring data would come from what GFDx describes as “compliance” data since it is based on information at the point of production. To avoid confusion with the general use of the term quality throughout this paper, the GFDx “quality” category will be referred to as “market and HH data”. The data presented below was obtained from the GFDx on 28 October 2020 and includes countries that have mandatory or voluntary fortification programs in place. 

For wheat flour fortification, 5 countries (Australia, Brazil, Chile, Mexico, and Peru) have “industry compliance data”. Liberia [21] and Senegal [22] also have industry compliance data for wheat flour obtained from authorized government entities, but are not included in the GFDx. Ten countries (Afghanistan, Brazil, Burkina Faso, Indonesia, Kazakhstan, Malawi, Nepal, South Africa, Tanzania, and Uganda) have “market and HH data” while 81 countries have data as reported by “expert opinion”. 

For maize flour, 2 countries (Brazil and Mexico) have “industry compliance data”, 5 countries (Malawi, Mexico, South Africa, Tanzania, Uganda) have “market or HH data”, and 16 countries (Brazil, Burundi, Costa Rica, El Salvador, Guatemala, Kenya, Malawi, Mozambique, Nigeria, Rwanda, South Africa, Tanzania, Uganda, United States, Venezuela, and Zimbabwe) have “expert opinion” data. 

For cooking oil, no countries have data obtained from “industry compliance data” or “expert opinion”. Nine countries (Bangladesh, Burkina Faso, Liberia, Malawi, Mozambique, Nigeria, Pakistan, Tanzania, and Uganda) have “market or HH data”. 

For rice, no countries have “industry compliance”, one country has “market or HH data” (Papua New Guinea) and 6 countries have “expert opinion” data (Costa Rica, Nicaragua, Panama, Philippines, Solomon Islands, and the United States). 

For salt, 7 countries have “industry compliance data” (Bangladesh, Colombia, Nigeria, Peru, Thailand, Uganda, and Uzbekistan), 23 countries have “market or HH data” (Afghanistan, Bosnia and Herzegovina, Burkina Faso, Cambodia, Eswatini, Ethiopia, Georgia, Indonesia, Kazakhstan, Kenya, Lao PDR, Lebanon, Liberia, Malawi, Morocco, Nepal, Papua New Guinea, Tajikistan, Tanzania, Tunisia, Uganda, Uzbekistan, and Zambia), and 4 countries (Colombia, Macedonia, Mexico, and Zimbabwe) have “expert opinion” data. Table 1 outlines countries with “compliance data” by staple food or condiment and how this data was collected. 

Table 1 illustrates that only a limited number of countries (a total of 14) have data on fortification compliance obtained by authorized government entities. Although there may be limitations to this data, namely countries with data that may not be included in the GFDx database that do, in fact, have compliance data, it points to an alarming trend globally. 

Rates of compliance, as reported in GFDx, ranged from 10–100% across all foods required to be fortified, pointing to a second problem of high variability in actual fortification compliance of the foods. Osendarp et al. also report that a review of external quality assurance activities from 25 countries, supported by the Global Alliance for Improved Nutrition (GAIN), found the percentage of foods meeting national standards ranged from 18% to 97%, with an average of 45% to 50% [13,23]. A study published in 2020, by Mkambula et al. [9], talks about the “quality gap” that exists in fortification programs. This gap refers to the difference between the proportion of households consuming a fortified food and those consuming a food fortified in accordance with the relevant national fortification standards. Data obtained by Mkambula et al. (2020) from 16 wheat flour surveys, 8 maize flour surveys, 14 oil and ghee surveys, and 21 salt surveys from multiple countries showed that of the 15% of households consuming fortifiable (i.e., industrially processed) wheat flour, 5% were adequately fortified; of the 22% of households consuming fortifiable maize flour, 3% were adequately fortified; of the 31% of households consuming fortifiable oil and ghee, 14% were adequately fortified; and of the 65% of households consuming fortifiable salt, 1% was adequately fortified. “Adequately fortified” for foods other than salt in the above study was estimated based on results where available in individual survey reports [9]. However, caution should be taken when interpreting these results, which are looking at adequately fortified products at the household level. Since there is a need to have data from point of production to infer compliance, this may over-estimate the quality gap due to potential fortificant losses in the food as it makes its way from the point of production to the market or household level because of varying storage and environmental conditions, and not as a result of industry non-compliance. 

Taken collectively, the information presented above does not necessarily infer food producers globally are fortifying foods inaccurately. It demonstrates that quality compliance data is hard to come by and, of the data that does exist, performance varies greatly with a trend towards poor performance. Without a national picture of compliance, there is no way to know whether or not fortified foods actually contain the correct amounts of vitamins and minerals per the national standard. Such a scenario risks programs being implemented ineffectively, wasting scarce resources, and jeopardizing their potential nutritional impact. Ideally, this picture of program performance would come from government monitoring data obtained from industry and not from outside development agencies for the sake of program ownership and sustainability. Although in lieu of government ability to collect this information, it is useful to have external stakeholder support to make timely program performance adjustments. The section below outlines challenges related to why this scenario of limited data and poor performance might exist. 

### 2.2. Documented Challenges Collecting and Acting Upon Regulatory Monitoring Data

Numerous studies have documented challenges that government inspectors and food producers face when collecting and acting upon data that ensures adherence to fortification standards.

Table 2 and Table 3 attempt to summarize what we know from 7 studies and 1 workshop between 2013 and 2020 [9,13,14,15,23,24,25,26]. Table 2 presents challenges faced by government inspectors collecting and using (e.g., collating, analyzing, reporting out on, and acting upon) regulatory monitoring information, while Table 3 presents challenges faced by industries trying to comply with national standards (both from an industry perspective and a government perspective). 

Table 2 and Table 3 also include findings from a Smarter Futures’ Fortification Monitoring ‘Challenge’ Workshop that took place virtually between August and October 2020 and that involved ten countries (Cameroon, Cote d’Ivoire, Ghana, Kenya, Mozambique, Nigeria, Senegal, South Africa, Tanzania, and Uganda). Teams from each country were asked to outline (a) their specific fortification monitoring challenges from the perspective of both industry and government and (b) how they might address these specific challenges using existing human and financial resources at their disposal. In other words, the solutions they were asked to propose to address their regulatory monitoring challenges could not include anything that required further inputs or resources. The goal was to spur creative thinking around how this data might be obtained in more effective and realistic ways today. The hope was that the teams would leverage multiple tools and methods that currently exist to address their challenges.

Unfortunately, most of the solutions obtained from country teams revolved around the need for additional training and additional funding. It is unclear whether the team responses were due to a lack of understanding of the question being asked, a lack of awareness of the tools and methods that currently exist to address their challenges, resistance internally to move away from more traditional means of collecting fortification quality data, or otherwise. This more nuanced understanding was not obtained from the workshop participants.

### 2.3. Global Resources to Address Pressing Monitoring Challenges 

There has been much effort over the years to provide resources to address the identified fortification monitoring challenges. Several resources have recently been developed at both the international and country level, with the goal of improving fortification program compliance. These include but are not limited to the 2018 Regulatory Monitoring of National Food Fortification Programs: A Policy Guidance Document, FortifyMIS, PalmaTrack, and the Micronutrient Fortification Index, each profiled below.

#### 2.3.1. Policy Guidance Document

In 2018, the Global Alliance for Improved Nutrition (GAIN) and Project Healthy Children (PHC) led the creation of Regulatory Monitoring of National Food Fortification Programs: A Policy Guidance Document [18]. The document was generated in response to recommendations that came out of the September 2015 Global Summit on Food Fortification, which culminated in the Arusha Statement on Food Fortification [27]. Among other things, the Statement set forth five recommendations for fortification in low- and middle-income countries, the second being to improve oversight (regulatory monitoring) and enforcement of food fortification regulations and standards. As a result, a regulatory monitoring working group was established to identify enabling factors that facilitate consistent regulatory monitoring practices and industry compliance. An outcome of the group’s efforts was the Regulatory Monitoring Policy Guidance Document. 

The document proposes a standardized systems-based approach for determining compliance built upon a foundation of realistic, feasible food fortification standards. It addresses common challenges faced by government regulatory agencies that are designated to monitor the fortification program and by food manufacturers as they seek to fortify appropriately. The document aims to reflect a consensus among fortification stakeholders, and to serve as a resource for those responsible for food fortification policy development and implementation. It is particularly aimed at those working in countries that have struggled to carry out regulatory monitoring activities on a consistent basis, and where the lack of compliance with fortification regulations and standards is an ongoing issue [18]. In addition to GAIN and PHC, the document was endorsed by the Iodine Global Network (IGN), the Food Fortification Initiative (FFI), and Technoserve. The text box below summarizes the highlighted recommendations from the policy guidance document. 

Highlighted recommendations from the 2018 Regulatory Monitoring of National Food Fortification Programs: A Policy Guidance Document [18]

1. Implement a standardized, realistic systems-based approach to determine compliance, emphasizing the process of fortification over regular testing of fortified food samples.
2. Develop a comprehensive audit checklist that covers food quality, food safety, and food fortification.
3. Use the premix reconciliation calculation to determine whether the manufacturing (fortification) process is sufficiently adding micronutrients to foods. This equation compares whether the amount of premix used correlates appropriately to the amount of fortified food produced over a set time period. Premix reconciliation is one task conducted during an audit at a food production site.
4. Within the country’s fortification standards, express each micronutrient specification as a target value encompassed by actionable limits.
5. Analyze composite samples of fortified foods quantitatively only periodically and as a means to validate the findings of an audit.
6. Implement a user-friendly, computerized management information system (MIS) to make the process of data collection, collation, analysis, interpretation, and results dissemination more efficient and effective.
7. Establish incentives that appeal to the food industry in addition to meaningful and enforceable penalties that drive consistent compliance among food manufacturers.
8. Involve non-traditional stakeholders in monitoring fortification programs at the commercial and household levels to extend resources and expand public engagement in the initiative.

#### 2.3.2. FortifyMIS

FortifyMISis an online data collection and aggregation approach for fortification monitoring developed by Project Healthy Children (PHC) and the Global Alliance for Improved Nutrition (GAIN). The Management Information System (MIS) provides an improved means for food producers and government inspectors to monitor the quality of fortified products while providing decision makers with timely information to improve program performance. The MIS can be used offline in the field and allows for strict confidentiality of information by users. Users include food producers, government inspectors, program managers, and laboratory staff, each with their own set of unique user and viewing privileges. The goals of FortifyMIS are to (1) simplify the process of compliance data collection for national-level food inspectors and food producers and (2) improve how food control agencies are informed of implementation challenges [28]. 

**Figure 1:**
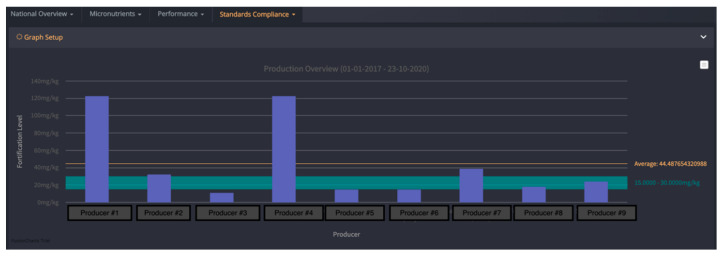
FortifyMIS dashboard data that displays how compliant cooking oil producers are to the national vitamin A standard over a select period of time compared to the national standard shown by the green horizontal bar (including minimum (15 mg/kg) and maximum (30 mg/kg) levels as designated in the national cooking oil fortification standard). Average oil fortification is 44 mg/kg; 6 producers are considered compliant since they fall above the minimum of 15 mg/kg. These data were collected by regulatory inspectors and can be viewed by program managers. Producer information has been masked for confidentiality.

**Figure 2:**
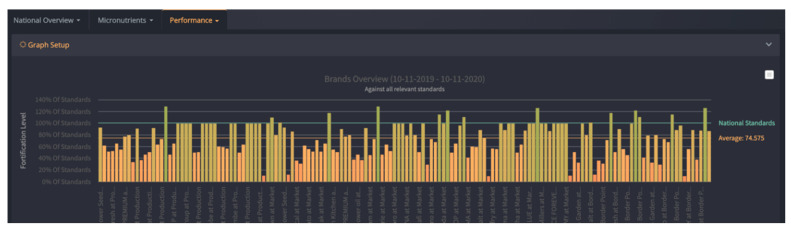
FortifyMIS dashboard data that displays brand performance for cooking oil against the national standard for vitamin A and indicates what percent of the standard is met across all data collection points (producer level, border points, and market level). Brand information has been masked for confidentiality.

*FortifyMIS* can be used on computers, tablets, and handheld mobile devices. It allows for automatic tracking of fortified food quality and safety data using customizable digital forms, real-time dashboards, and tailored data reporting methods. The platform aims to reduce the time and cost of monitoring and improve overall program performance by quickly tracking the quality of foods and identifying where improvements are needed. One of the biggest challenges, outlined above, that the MIS addresses is the need for a central database to collate collected information, and the need to streamline data collection for regulatory inspectors. FortifyMIS is currently being piloted in Bangladesh, Mozambique, Nigeria, Pakistan, and Tanzania. Examples of data outputs from the MIS are provided in Figure 1 and Figure 2 with producer and brand data intentionally hidden for confidentiality.

#### 2.3.3. PalmATrack

**Figure 3:**
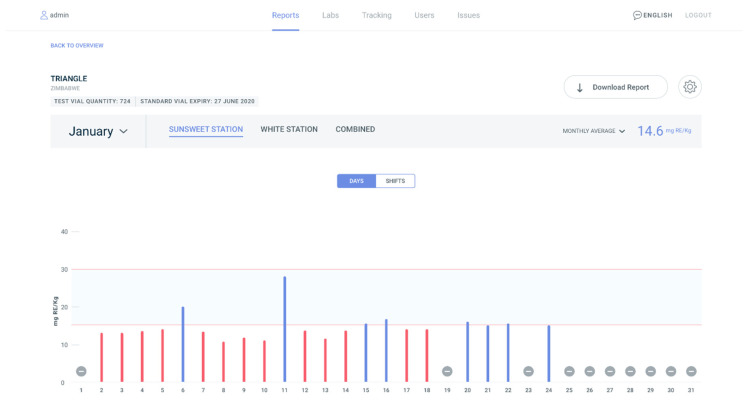
PalmATrack industry dashboard for the month of January for one sugar producer that has two production facilities (Sunsweet Station and White Station). The graph displays iCheck test results for vitamin A in the sugar. The x axis displays the days of the month and the y axis displays the test results for vitamin A in milligrams of Retinal Equivalent (RE) per kilogram. Red bars indicate test results that are below the national standard and blue bars indicate test results that within the national standard. Dashes indicate no test results for that day.

Developed in 2018 by Millhouse, a supplier of vitamin A palmitate and fortified sugar for food industries in Africa, PalmATrack is an online platform designed to capture live production data from sugar industries. It is a platform that empowers mills, laboratories, governments, and related agencies in assessing vitamin A quality in line with government fortification regulations. PalmATrack was developed to track and display vitamin A iCheck results, which provide nutrient levels in fortified foods. This is a key indicator to inform industry about regulatory compliance with fortification standards. Users include laboratory staff, mill staff, and government, each with their own set of data viewing privileges. PalmATrack allows for remote monitoring so Millhouse can view progress and anticipate bottlenecks to support their partner industries. PalmATrack is supporting monitoring within sugar production facilities in Mozambique, Zimbabwe, and Malawi, with plans to scale to the maize industry throughout Africa [29]. Figure 3 illustrates an example PalmATrack dashboard as viewed by industry for the month of January. This industry user has two sugar production facilities (Sunsweet Station and White Station) that are displayed across the top of the graph.

#### 2.3.4. Micronutrient Fortification Index

With the goal of seeking a cost-effective strategy that provides a commercial incentive for processors to comply with food fortification standards in Nigeria, Technoserve’s Strengthening African Processors of Fortified Foods (SAPFF) program conceptualized and developed the Micronutrient Fortification Index (MFI). MFI supports existing regulatory systems with an industry-driven initiative that effectively differentiates between compliant and non-compliant companies by the extent to which they meet industry benchmarks, including compliance, informed by Nigerian standards. At the core of the MIF are three weighted components which feed into a score: a self-assessment, product testing, and external verification of the self-assessment. Companies’ overall scores are presented on a dashboard, which is updated annually to show progress and gaps, ultimately contributing to an industry-wide platform emphasizing quality standards as a key performance indicator [30]. 

Although these resources address many of the challenges outlined in Table 2 and Table 3, their use and rollout across country programs has been limited.

## 3. Use of Premix Reconciliation to Infer Compliance

Although there is no one solution to address the myriad of challenges that industry and regulatory inspectors face when assessing the quality of fortified foods, there is one practice that appears consistently under-utilized that could dramatically reduce the burden of assessing quality, both at the industry and regulatory inspector levels. 

The premix reconciliation calculation is a back-of-the-envelope calculation that allows a food producer or regulatory inspector to infer compliance to a national standard based on two pieces of information: the quantity of premix used and the quantity of food produced over the same period of time. The calculation, outlined in Table 4, is a recommendation highlighted in the 2018 Regulatory Monitoring Policy Guidance Document and incorporated into the FortifyMIS and is currently being used in several countries some more successfully than others; these countries will be outlined below. It is a practice that most industry personnel are familiar with, as it is often a regular part of quality assurance practices, however, it is not regularly shared with external inspectors either because it is not asked for by the inspectors during industry audits, or because there is a lack of trust between government inspectors and food producers. This lack of trust may be due to a variety of factors, including use of data by government inspectors for reasons other than disclosed, such as for industry taxation estimation schemes.

Using this calculation as part of an industry audit addresses most of the challenges outlined in Table 2 and Table 3 under laboratory testing and equipment and, to some extent, challenges related to budget, human resources, and data collection. Using this calculation method precludes the need for regular quantitative testing, which although important to occasionally verify compliance, is wrought with issues including lack of training on appropriate sampling procedures to ensure meaningful results, measurement error and poor understanding of measurement uncertainty and how this relates to interpretation of results, inability to successfully and reliably detect small quantities of specific nutrients (e.g., folic acid), poor capacity to transport collected samples to a functional laboratory, poor capacity of trained laboratory personnel who practice the testing methods regularly, lack of access to and ability to purchase testing reagents, lack of a meaningful turnaround time that enables industry to make quality adjustments if required, and the lack of a recuring line in the budget of industry or government. 

Additionally, although fortification monitoring is a part of food process control procedures, it generally falls low on the priority list (since food safety takes precedence, as it should, over food quality). Therefore, we need to be conscious of what countries are being asked to do when it comes to assessing fortification quality. The protocols need to be quick, nimble, accurate, realistic for the context, and effectively able to balance safety and quality. 

Another benefit of using the premix reconciliation method of inferring compliance is that it is not built on the assumption that food safety inspections are effectively overseeing fortification compliance within a country. Although it is best practice to incorporate fortification audits and inspections into already-existing government agency food safety inspections and protocols [18], this assumes an active food inspection system is in place and practiced regularly, and it also assumes that the up-front training required for inspectors to conduct audits can take place. Food inspection capacity and operations are limited in many countries due to a lack of human and financial resources to deploy food inspectors, unclear or non-existent mandates for food inspectors, numerous competing priorities for inspection, and other factors. Where food safety inspections do not occur or where there are no food safety inspectors, alternative means of collecting compliance information, which do not rely on government inspections, can use this calculation effectively.

A good example of this is in South Africa. Due to competing demands faced by food safety inspectors (in South Africa, these are Environmental Health Officers) and the fact that food safety inspectors do not have a mandate to assess fortification compliance in the current decentralized environment (e.g., the national government can ‘request’ not ‘instruct’ provinces to conduct an inspection and provinces can ‘request’ not ‘instruct’ municipalities to do the same) [31], the Department of Health is exploring an alternative means of collecting compliance information through an already-existing data collection system, the South African Grain Information Service (SAGIS). SAGIS in a non-profit, independent data collection system with the goal of gathering, processing, analyzing and timely distribution of reliable agronomic information to key stakeholders. SAGIS also renders additional information services to industries, for example the monitoring of import tariffs, audit certificates for minimum market access, and weekly import and export figures for the maize, wheat, sorghum, and oilseeds industries [32]. Currently, food producers in South Africa report production figures to SAGIS on a monthly basis and there is a level of trust that exists between producers and SAGIS enabling this data exchange to happen. With the inclusion of an additional data point, namely premix usage over that same period of time, fortification compliance could be inferred using the premix reconciliation calculation without having to deploy inspectors regularly to each producer. Instead, struggling producers could be identified through this process and targeted for support visits to assist with practices and procedures. Discussions are ongoing with SAGIS regarding the collection of this additional data point to infer national compliance [33].

Other national programs using the premix reconciliation method at the government level to monitor compliance include Australia (potentially), Jordan, and Peru. Based on data compiled in GFDx, Australia combines quality audits of mills with samples taken for quantitative testing. It is unclear, however, whether or not Australian food control authorities use this calculation during mill audits or whether this calculation is part of the internal quality assurance procedures that inspectors assess. Additionally, based on data reported in GFDx, Peru conducts premix verification calculations in a subset of mills, although they rely primarily on quantitative testing. 

Jordan is a very clear example of where this method is consistently employed. In 2009, Jordan’s fortification program began collecting data on: (1) monthly production of flour (obtained from mill production records) and (2) the number of boxes of premix used in the past month (obtained from premix storage logs) in order to streamline their monitoring process. A relatively limited number of staff were required to calculate the average addition rate and the percent of the target addition rate to infer mill-specific compliance while simultaneously providing an aggregate picture of national program performance. Samples were also tested quantitatively for iron as a means of verifying the findings [34]. Jordan does not appear as a country with compliance data in the GFDx because the information is not publicized in reports. 

Other countries that have attempted to use the premix reconciliation method to infer compliance at a national level include Afghanistan, Bangladesh, Kenya, Nigeria, Tanzania, and Zimbabwe but implementation in these countries has had limited success. The reasons for this include, but are not limited to: industry sharing the data with development partners but not with government (Afghanistan, Bangladesh); industry not sharing the data with government because of a lack of trust between industry and government (Nigeria); a disjointed government regulatory structure that would facilitate obtaining this data (Kenya, Tanzania), and no single agency to which all of the collected data comes to that is responsible for collating and acting upon the results (Afghanistan, Bangladesh, Kenya, Nigeria, Tanzania, Zimbabwe) [31]. Nigeria is using a unique industry-wide self-monitoring approach to collect this data through the formation of fortification subcommittees. These sub-committees are industry-specific (for example there is a wheat flour subcommittee and a cooking oil sub-committee) and each serve as an industry wide monitoring mechanism allowing industry to check industry and ensure compliance is being met. However, this information is not shared openly with government [15]. Nigeria is also using TechnoServe’s Micronutrient Fortification Index which has been discussed above. Zimbabwe has successfully incorporated the premix reconciliation method into their regulatory monitoring protocols but have struggled to get fortification effectively off the ground due to other challenges, namely premix sourcing, so it is not yet clear how effective this method will be [31].

So why is this method of data collection not more widely used in fortification programs? Inferring compliance by means of data from the premix reconciliation calculation assumes two things: (1) there is a means of collecting this information from industry (e.g., from existing internal production records, calculated by regulatory inspectors during audits, or obtained automatically from the mill’s dosifier where such technology exists) and (2) there is a means of collating, sharing, and acting upon the information collected. The Sanku project in Tanzania is currently using “smart dosifiers” that record operational data such as hours and days of operation, amount of grain milled, and amount of premix used among small-scale maize mills [35]. Is it worth exploring the use of similar technology at the large-scale production level to offset external monitoring challenges? Where there is no automated technology there needs to be (a) an inspector collecting this information during a production audit and ensuring it goes to a central location or (b) a producer sharing this information with a central database, requiring either food safety inspectors with a mandate to collect this information or a central database that exists, that is functional, and that industry can reliably use and trust won’t be used for purposes other than understanding fortification quality. 

## 4. Discussion

The countries that have been able to obtain a consistent picture of national fortification compliance, mentioned above, represent “positive deviants” of fortification monitoring because they have been able to successfully obtain nationally recorded data on fortification quality while facing similar (although not identical) challenges as other countries. The central premise of positive deviants [36,37,38] is that solutions to problems (faced by individuals, agencies, or communities) often already exist within that community, and that certain individuals in the community, who have access to the same resources, are able to solve the problem in ways that can be generalized to other members and effectively adopted and sustained [39]. The solutions employed by positive deviants then, are based on successful practices within the community rather than more theoretical concepts [38]. Much can be learned from these positive deviants.

Yet, there is still a limited number of countries collecting quality data and demonstrating compliance, and few countries using the tools and resources that have been developed to address these specific problems. This points to the need for greater communication and dissemination of existing resources and tools, resources to train individuals in these tools, and greater alignment of the recommendations and tools to existing challenges. It also highlights the fact that there may be more nuanced issues that none of the existing resources and tools currently address. This may include factors already discussed here such as trust between government and industry or the need for an intermediary body to collate compliance data in place of regulatory food safety inspectors (such as is proposed in South Africa). Alternatively, it may include other factors such as the need for more effective business models and more realistic incentives for industries to follow when it comes to ensuring fortification quality, the realities of introducing online technology to collect data when a reliance on paper forms has been the norm for so long, or the difficulties of changing how national food control agencies operate or how national food safety inspections take place within the complexities of governments mired in politics and bureaucracy. 

This paper purposely addresses suggested means of improving fortification quality data collection and does not address the legal structures that may need to be in place for monitoring quality in both voluntary and mandatory fortification programs, nor does it outline the details of how to design a regulatory monitoring framework for fortification programs. These are important design elements that will affect how and which foods are fortified and effectively assessed for quality. As noted earlier, a limitation to the analysis presented in this paper is the fact that the GFDx database may not include all countries that do, in fact, have compliance data. Where additional information outside of GFDx could be obtained (e.g., for Liberia and Senegal), this was included. 

In short, this paper leverages the experiences of strong performing programs in addition to lessons learned around what has not worked across programs and proposes a practical way forward that countries can use to address the fortification “quality gap”:

- **Use premix reconciliation to infer compliance.** Incorporate premix reconciliation into industry food safety and fortification audits. Follow the 2018 Global Regulatory Monitoring Policy Guidance Document recommendations regarding less frequent quantitative testing only as a means of verifying industry audits, potentially only once or twice a year.
- **Leverage producer associations or similar neutral bodies.** If a country does not have functioning food safety processes into which the premix reconciliation calculation can be incorporated or does not have enough human or financial resources to deploy inspectors, seek an alternative means of collecting the two key data points: production data and premix usage over the same period of time from industry. This may be via producer associations, subcommittees, or already-existing databases within the Ministry of Finance, Ministry of Health, or Ministry of Trade and Customs that are trusted by industry.
- **Cultivate trust through open discussions with producers/processors.** Discuss openly with producers and, importantly, producer associations where they exist, how these two data points could be obtained and shared in a way that both parties (government and producers) are comfortable with. Discuss what would cultivate a more trusting relationship between producers and government that would allow government to provide greater support to industry in their fortification efforts (e.g., what incentives would be most meaningful to them, what would timely audit and inspection feedback look like, etc.), and what would allow both parties to work towards generating a national picture of fortification compliance.

**Assumptions that should be avoided:**

- Food safety inspections take place and can effectively absorb fortification activities.
- Regular and rigorous laboratory testing should be the primary means by which to determine compliance.
- Industry will automatically be willing to share data with government inspectors without first building a relationship of trust.
- Government inspectors can reliably communicate results back to producers and processors in time to correct production practices before a product reaches the market.
- The only way to understand industry adherence to national fortification standards is through government inspectors.
- Viewing fortification programming through the lens of the public sector is more effective than through the lens of the private sector.
- Relying on government policing is more effective that private sector support.
- Low- and middle-income countries can accommodate fortification quality protocols that are more stringent than what most developed countries practice.

However, there is no one size fits all approach to the fortification quality gap challenge. Where some countries are able to find solutions, others will not. This is an area that is in need of a true understanding of what has worked in other settings, coupled with creative strategic approaches that work in each unique setting. The effective implementation of any new solution will be dependent on how well it is tailored to the country context. 

## 5. Conclusions

This paper outlines current global challenges faced by food producers and government regulatory inspectors in obtaining data pertaining to the quality of fortified food produced in national fortification programs, resources that have been developed to address these challenges, “positive deviant” countries that have overcome challenges, and where we stand today in terms of being able to quantify effective implementation. It makes the argument that, although imperfect, there is an under-utilized but potentially effective means of obtaining a fairly accurate picture of producer-specific and national program compliance: the premix reconciliation calculation. Although it relies on a level of trust between industry and government, it addresses many of the documented monitoring challenges and allows programs to employ an effective means of data collection using, in most cases, existing human and financial resources. Its implementation, however, must take into account the unique and nuanced challenges that are faced at a country level, particularly around data sharing, if it is to be a successful and sustained approach.

## Figures and Tables

**Table 1:** Data from GFDx (unless otherwise indicated) outlining countries with mandatory or voluntary fortification programs in place that have “compliance data”, defined as industry compliance data obtained by authorized government entities on production volumes, market share, or samples and facilities monitored, and the methods used to obtain the data.

Country	Timeframe	Staple Food/Condiment	Reported Compliance Range	Methods
Australia	2010–2011	Wheat flour	Unspecified	“Mills were audited by food control authorities to determine if they had quality assurance procedures in place and one wheat flour sample was taken per mill and analyzed in a lab for folic acid. All mills had quality assurance procedures in place and were deemed to be in compliance with the standard including five mills whose folic acid levels in samples were outside of the uncertainty range.” This was likely due to the challenges reliably detecting small quantities of this nutrient.
Brazil	2006, 2007, 2010, 2011, 2012	Wheat flour	73–93%	“Wheat flour samples from mills were analyzed quantitatively for iron.”
	2006, 2008, 2010, 2011, 2012	Maize flour	16–100%	“Maize flour samples from mills were analyzed quantitatively for iron.”
Chile	2007–2014 (excluding 2013)	Wheat flour	10–90%	“Flour samples were taken from national millers and imports four times a year. Samples were measured for thiamin, riboflavin, folic acid, and iron levels.”
Mexico	2010–2017	Wheat flour	70–100%	“Government report of samples taken from mills. Samples measured for iron, folic acid, and zinc levels. Results reflect the lowest proportion of samples that met nutrient levels in the fortification standards.”
	2010–2017	Maize flour	Not included	“Data obtained from a government report of samples taken from mills. The method is not detailed. Results reflect proportion of samples that met folic acid levels in the standard.”
Peru	2009–2017	Wheat flour	50–100%	“Government inspectors took flour samples from mills. Thiamine, riboflavin, niacin, folic acid, and iron levels were measured. Results reported use production volumes to generate the percent of flour compliant based on laboratory analyses. Premix reconciliation calculations were completed in a subset of mills.”
Liberia *	2014–2020	Wheat flour	23–100%	Government inspectors take wheat flour samples from mills, import sites, and markets and test for iron using iCheck equipment.
	2014–2020	Cooking oil	6–83%	Government inspectors take cooking oil samples from import sites and markets and test for vitamin A using iCheck equipment.
		Salt	21–100%	Government inspectors take salt samples from import sites and market and use the titration method to determine iodine content.
Senegal **	2018–2019	Wheat flour	90%	Wheat flour samples from mills are quantitatively analyzed at the control laboratory of the Ministry of Commerce.
Bangladesh	2006–2018	Salt	79–97%	“Based on samples collected from production and tested for iodine.”
Colombia	2014–2015	Salt	60–62%	“Based on samples collected at production level and tested by an authorized national lab for iodine and fluoride levels.”
Nigeria	2011–2019	Salt	67–98%	“Based on quantitative tests of samples taken from production level. In 2018–2019, the USI/IDD Taskforce was set up in response to the decline in the compliance level of salt iodization at the household level in 2013. The Standards Organization of Nigeria (SON) authorized the release of the annual compliance data since the Taskforce started monitoring at the factory level. Data reported are the aggregate of onsite sampling assessment data collected by staff of SON during unscheduled monthly visits (January–December every year) to the two salt processing companies currently in operation.”
Peru	2009–2010	Salt	41–59%	“Based on samples collected.”
Thailand	2011–2018	Salt	13–92%	“Based on sample collection data from the Thai Food and Drug Administration (FDA).”
Uganda	2018	Salt	79%	“Samples collected from production and tested for iodine content.”
Uzbekistan	2004 and 2014	Salt	39–75%	“Data source unknown.”

Source: Global Fortification Data Exchange. Accessed on 28 October 2020. (http://www.fortificationdata.org) [20]; * Source: Mambu, S. (National Standards Laboratory, Monrovia, Liberia). Personal communication, 2020 [21]; ** Source: Ndao, I. (Nutrition International, Dakar, Senegal). Personal communication, 2020 [22].

**Table 2:** Challenges faced by government regulatory agencies (from the perspective of government delegates themselves, food producers, and external stakeholders) when monitoring food fortification categorized by ‘challenge type’ [9,13,14,15,23,24,25,26].

Challenge Type	Government Regulatory Agency Challenges
Regulations/Government Structure	Need for clear regulations that identify the roles and responsibilities of stakeholders and how they should collaborate
	Regulations related to monitoring, inspection, and enforcement are often fragmented and not appropriately embedded within legal frameworks, leading to a lack of (or weak) enforcement
	Need for improved regulatory agency structure
	Poor government coordination
	Need for better harmonization and integration of fortification efforts vertically into food safety mechanisms as a subset of food control within the appropriate national entities
	Lack of clear specifications and objective assignment of enforcement mechanisms and penalties in the legal framework
	Ongoing reorganization of regulatory agencies
	Fragmented system for collecting data/agency/inspector overlap
Enforcement	Low priority and capacity for enforcement
	Lack of willingness on the part of government inspectors to take on the “political risk” of enforcement (e.g., using penalties was seen as politically risky due to perceived or real resistance from interest groups or mill associations) and often lead to penalties that were not serve enough to encourage adequate fortification
Laboratory/Testing/Equipment	Laboratory capacity constraints, both in terms of trained analysts and local availability/cost of equipment and reagents
	Both regulators and industry have an over-reliance on end-product quantitative testing and lab results, which have a high margin of error and are time consuming to conduct
	Lack of reliance on industry audits in place of quantitative testing
	Need for food inspectors to consider laboratory analysis in combination with additional critical information, including information obtained from mill or factory inspections
	Lack of cost-effective and field-friendly tools to ensure quality to assist with effective enforcement of fortification legislation; cost and availability of reagents
	Quantitative testing of multiple micronutrients as opposed to one market nutrient and/or testing of micronutrients that are considerably difficult to measure because they are labile, added in minute quantities, or have a wide margin of error leading to high investment of lab resources and often unreliable results but often results that industry must respond to
Budget	Need for improved regulatory agency financing
	Limited national budget allocations for fortification
Human Resources	Need for improved regulatory agency capacity
	Lack of trained inspectors and analysts (for product sampling and laboratory testing)
	Limited personnel for legal action
	Lack of motivation at the implementing local government level
	Corruption among inspection personnel
	Delay in getting results to millers
Data collection	Over-reliance on monitoring at the retail level in place of producer or import level
	Lack of a centralized data collection mechanisms for collected data
	Need to simply the process of regulatory monitoring data collection for inspectors
	Fragmented system for collecting data/agency/inspector overlap
Relationship between public and private sectors	Lack of trust between government and industry

**Table 3:** Challenges faced by industry (food producers) (from the perspective of food producers, government inspectors, and external stakeholders) ensuring the quality production of fortified foods categorized by ‘challenge type’ [9,13,14,15,23,24,25,26].

Challenge Type	Industry (Food Producer)
Regulations	Need for clear regulations that identify the roles and responsibilities of stakeholders and how they should collaborate
Enforcement	Competition with non-compliant producers
	Lack of realistic, meaningful, and consistent enforcement (incentives and penalties)
Laboratory	Poor laboratory capacity
	Lack of locally available reagents
Equipment	Lack of fortification equipment that is locally available, accessible, and affordable
Premix	High price of premix
	Poor quality premix
	Lack of duty-free premix
Budget	Lack of internal budgets that include fortification
Human Resources	Lack of training
	Need for training on internal monitoring with a special focus on process control
	Lack of awareness of standards
	Delay in results to millers
Relationship between public and private sectors	Need for communication between sectors (e.g., industry and regulatory agency)
	Lack of trust between government and industry
Consumers	Lack of product market demand
	Lack of accountability to consumers
Other	Lack of an effective business model for fortification
	Purposeful under-fortification

**Table 4:** Data points and calculations required for the premix reconciliation calculation used to infer fortification compliance with national standards.

Item *	Unit	Where to Locate
A. Starting inventory of premix	MT **	See facility records
B. Amount of premix purchased	MT	See facility records
C. Ending inventory of premix	MT	See facility records
D. Amount of premix used	MT	Calculate: A + B − C
E. Fortified product produced	MT	See facility records
F. Actual premix addition rate	g ***/MT	Calculate: D/E × 1000
G. Target premix addition rate	g/MT	Provided by premix producer
Result: Percent of target addition rate achieved	%	Calculate: F/G × 100

* The same period of time should be used for A–E; ** Metric Tons; *** Grams. Source: 2018 Regulatory Monitoring of National Food Fortification Programs: A Policy Guidance Document [18].

## References

[1] (2016). Global Panel of Agriculture and Food Systems for Nutrition. https://www.glopan.org.

[2] Horton S., Pettifor J.M., Zlotkin S. (2004). The economic impact of micronutrient deficiencies. Micronutrient Deficiencies During the Weaning Period and the First Years of Life.

[3] Black M.M. (2003). Micronutrient deficiencies and cognitive functioning. J. Nutr..

[4] Hoddinott J., Rosegrant M., Torero M., Lomborg B. (2013). Hunger and malnutrition. Global Problems, Smart Solutions: Costs and Benefits.

[5] Horton S. (2006). The economics of food fortification. J. Nutr..

[6] Baltussen R., Knai C., Sharan M. (2004). Iron fortification and iron supplementation are cost-effective interventions to reduce iron deficiency in four subregions of the world. J. Nutr..

[7] Beal T., Massiot E., Arsenault J.E., Smith M.R., Hijmans R.J. (2017). Global trends in dietary micronutrient supplies and estimated prevalence of inadequate intakes. PLoS ONE.

[8] Keats E.C., Neufeld L.M., Garrett G.S., Mbuya M.N.N., Bhutta Z.A. (2019). Improved micronutrient status and health outcomes in low- and middle-income countries following large-scale fortification: Evidence from a systematic review and meta-analysis. Am. J. Clin. Nutr..

[9] Mkambula P., Mduduzi M.N.N., Rowe L.A., Sablah M., Friesen V.M., Chadha M., Osei A.K., Ringholz C., Vasta F.C., Gorstein J. (2020). The unfinished agenda for food fortification in low and middle-income countries: Quantifying progress, gaps and potential solutions. Nutrients.

[10] Vijaya K., Chadha M., Rowe L.A., Thompson A., Jain S., Walters D., Martinez H. (2020). Reducing the burden of anemia and neural tube defects in low- and middle-income countries: An analysis to identify countries with an immediate potential to benefit from large-scale mandatory fortification of wheat flour and rice. Nutrients.

[11] Dary O., Mora J.O. (2002). Food fortification to reduce vitamin a deficiency: International Vitamin A consultative group recommendations. J. Nutr..

[12] Horton S., Ross J. (2003). The economics of iron deficiency. Food Policy.

[13] Luthringer C.L., Rowe L.A., Vossenaar M., Garrett G.S. (2015). Regulatory monitoring of fortified foods: Identifying barriers and good practices. Glob. Health.

[14] Rowe L.A., Garrett G.S., Luthringer C.L., Pachón H., Verster A. Summit recommendation 2: Regulatory monitoring. Sight and Life: 36–39 April 2016. http://www.sightandlife.org/fileadmin/data/Magazine/2016/Suppl_to_1_2016/FutureFortified.pdf.

[15] Fortification Monitoring “Challenge” Workshop Smarter Futures and Lodestar Center of Excellence Workshop Proceedings. October 2020. https://static1.squarespace.com/static/5e1df234eef02705f5446453/t/5f3d65b4ea586222105e3f7b/1597859255601/2018_SADC_Workshop-Report_v2.pdf.

[16] Akhigbe O., Amyot D., Reichards G.S. (2016). Monitoring and management of regulatory compliance: A literature review. Int. J. Inf. Process. Manag..

[17] Bittisnich D. (2020). Food Fortification Regulatory Monitoring Assessment.

[18] Global Alliance for Improved Nutrition (GAIN), Project Healthy Children (PHC) (2018). Regulatory Monitoring of National Food Fortification Programs: A Policy Guidance Document. Global Fortification Technical Advisory Group (GF-TAG). https://s3-us-west-2.amazonaws.com/gfdx-publishefiles/Regulatory+Monitoring+Policy+Guidance+April+2018.pdf.

[19] Allen L., de Benoist B., Dary O., Hurrel R., World Health Organization (WHO), Food and Agriculture Organization (FAO) (2006). Guidelines on Food Fortification with Micronutrients.

[20] Global Fortification Data Exchange (GFDx). http://www.fortificationdata.org.

[21] Mambu S. (2020). Personal communication.

[22] Ndao I. (2020). Personal communication.

[23] Osendarp S.J., Martinez H., Garrett G.S., Neufeld L.M., De-Regil L.M., Vossenaar M. (2018). Large-scale food fortification and biofortification in low-and middle-income countries: A review of programs, trends, challenges, and evidence gaps. Food Nutr. Bull..

[24] Van den Wijngaart A., Begin F., Codling K., Randall P., Johnson Q.W. (2013). Regulatory monitoring systems of fortified salt and wheat flour in selected ASEAN countries. Food Nutr. Bull..

[25] Dijkhuizen M.A., Wieringa F.T., Soekarjo D., Van K.T., Laillou A. (2013). Legal framework for food fortification: Examples from Vietnam and Indonesia. Food Nutr. Bull..

[26] Gayer J., Smith G. (2015). Micronutrient fortification of food in Southeast Asia: Recommendations from an expert workshop. Nutrients.

[27] (2015). The Arusha Statement on Food Fortification. https://www.fantaproject.org/sites/default/files/Final-Arusha-Statement-on-Food-Fortification-Sep2015.pdf.

[28] The Food Fortification Initiative. https://www.ffinetwork.org/tools#fortificationmonitoring.

[29] Dove M. (2020). Personal communication.

[30] Durotoye T. (2020). Personal communication.

[31] Randal P. (2020). Personal communication.

[32] South Africa Grain Information Service. https://www.sagis.org.za/index.html.

[33] de Hoop M. (2020). Personal communication.

[34] Wirth J.P., Nichols E., Mas’d H., Barham R., Johnson Q.W., Serdula M. (2013). External mill monitoring of wheat flour fortification programs. An approach for program managers using experiences in Jordan. Nutrients.

[35] Mildon A., Klaas N., OʹLeary M., Yiannakis M. (2015). Can fortification be implemented in rural African communities where micronutrient deficiencies are greatest? Lessons from projects in Malawi, Tanzania, and Senegal. Food Nutr. Bull..

[36] Marsh D.R., Schroeder D.G., Dearden K.A., Sternin J., Sternin M. (2004). The power of positive deviance. BMJ.

[37] Sternin J., Choo R. (2000). The power of positive deviancy. An effort to reduce malnutrition in Vietnam offers an important lesson about managing change. Harv. Bus. Rev..

[38] Bradley E.H., Curry L.A., Ramanadhan S., Rowe L.A., Nembhard I.M., Krumholz H.M. (2009). Research in action: Using positive deviance to improve quality of health care. Implement Sci..

[39] Walker L.O., Sterling B.S., Hoke M.M., Dearden K.A. (2007). Applying the concept of positive deviance to public health data: A tool for reducing health disparities. Public Health Nurs..

